# *Hedyotis diffusa*–*Sculellaria barbata* (HD–SB) suppresses the progression of colorectal cancer cells via the hsa_circ_0039933/hsa-miR-204-5p/wnt11 axis

**DOI:** 10.1038/s41598-023-40393-1

**Published:** 2023-08-16

**Authors:** Danye Zhu, Shanmin Yuan, Cong Chen

**Affiliations:** 1 Department of TCM Rehabilitation Medicine, Guangzhou Dongsheng Hospital, Guangzhou, 510000 Guangdong China; 2 Department of Traditional Chinese Medicine, Ganzhou People’s Hospital, Ganzhou, 341000 Jiangxi China

**Keywords:** Biochemistry, Cell biology

## Abstract

Our previous study confirmed that the combination of *Hedyotis diffusa* (HD) and *Scutellaria barbata* (SB) significantly inhibited colorectal cancer cell proliferation and the WNT signaling pathway. However, the exact molecular modulation remains unclear. In this study, colorectal cancer cells (SW620) were treated with 1 mg/mL HD–SB for 24 h, and high-throughput sequencing of circRNAs was performed. The level of hsa_circ_0039933 in three colorectal cancer cell lines (HT-29, SW620, and HCT116) was verified by qPCR. After transfection of hsa_circ_0039933 overexpression plasmids or small interfering RNAs, CCK8, apoptosis, cell migration, and cell invasion were utilized to evaluate the function of hsa_circ_0039933 in the progression of colorectal cancer cells. We identified hsa_circ_0039933, which was downregulated in HD–SB-induced colorectal cancer cells and positively related to colorectal cancer progression. In SW620 cells with relatively high expression of hsa_circ_0039933, interfering with the expression of hsa_circ_0039933 inhibited the proliferation, invasion, and migration of SW620 cells. In HCT116 cells with relatively low expression of hsa_circ_0039933, overexpression of hsa_circ_0039933 promoted the proliferation and invasion and migration ability of HCT116. Mechanistically, hsa_circ_0039933 targeted hsa-miR-204-5p to increase the expression of wnt11, leading to the activation of the Wnt pathway, thereby promoting the proliferation of colorectal cancer cells. This work revealed the potential molecular mechanism of HD–SB for the treatment of colorectal cancer, which was to inhibit the Wnt signaling pathway through the hsa_circ_0039933/hsa-miR-204-5p/wnt11 axis, then suppressing proliferation, migration, and invasion in the colorectal cancer cell.

## Introduction

Colorectal cancer (CRC) is the fourth most common malignant tumor in the world, with about 1.2 million new cases and 0.9 million deaths each year, accounting for 10% of all cancers and cancer-related deaths diagnosed worldwide, seriously threatening human life^1,2^. Since the early stage of CRC is an asymptomatic disease, most patients are usually diagnosed with advanced CRC at diagnosis^3^. In addition to the aging population and poor dietary habits, adverse risk factors such as obesity, smoking and alcohol consumption also increase the risk of CRC^4,5^. With the development of cancer biomarkers, laparoscopic surgery, molecular targets and immunotherapy^6–8^, the treatment of CRC has gradually diversified, but the overall survival of CRC patients remains unsatisfactory, and there is an urgent need to seek new and effective treatments.

Traditional Chinese medicine (TCM) has been applied for hundreds of years, gradually becoming an integral part of comprehensive treatment for cancers^9^. TCM has been shown to enhance the therapeutic effects of chemotherapy and radiotherapy in the treatment of colorectal cancer^10,11^. *Hedyotis diffusa* (HD) belongs to the genus Auricularia of Rubiaceae, which is mostly distributed in tropical areas^12^. It is widely used as a detoxifying herb in TCM due to its functions of clearing heat, removing toxicity, activating blood and reducing swelling^13,14^. Accumulating evidence has proved that HD has anticancer activity in the treatment of both breast cancer and cervical cancer^15,16^. HD has been reported to be able to inhibit colorectal cancer growth in vivo and in vitro^17^. *Scutellaria barbata* (SB) belongs to the Labiatae family, which has improved cellular immune function and anticancer effects and is widely applied to treat various tumors^18^. A previous study demonstrated that SB extract inhibited tumor growth of prostate cancer via PI3K/Akt pathway^19^. HD–SB in compatibility with TCM is applied in clinical cancer treatment in the form of couplet medicine. HD–SB was confirmed to decrease Akt activation by decreasing the expression of miR-155, leading to bladder cancer cell apoptosis^20^. Our previous study revealed that HD–SB could inhibit the proliferation of CRC cells, but its specific molecular mechanism remains to be elucidated^21^.

CircRNAs are a type of non-coding RNA, which are circular RNA molecules formed by covalent connection and closure of upstream/downstream splice sites^22^. The covalently closed loop structure enables circRNAs with high stability, making them promising biomarkers for cancer diagnosis and treatment^23^. CircRNAs have been demonstrated to exert biological functions in cancers by acting as microRNA sponges^24,25^. Numerous studies have confirmed that aberrant expression of circRNAs is closely related to several diseases including CRC. It was identified that circ-ERBIN was elevated in CRC, and it promotes colorectal cancer growth and metastasis by mediating HIF-1α through miR-125a-5p and miR-138-5p, suggesting that circ-ERBIN is a potential target for CRC treatment^26^. CircCDC6, a tumor suppressor, was downregulated in CRC patient tumor tissues, where it inhibited tumor growth and glycolytic metabolism in CRC via the mir-3187-3p/PRKAA2 axis^27^. The hallmark wnt signaling pathway is an integral signaling pathway in CRC^28^. A recent study indicates that hsa_circRNA_104348 acts as a competitive endogenous RNA (ceRNA) that promotes hepatocellular carcinoma progression by activating the wnt/β-catenin pathway^29^. CircRNA_100367 attenuates esophageal squamous cell carcinoma proliferation, migration and radiation resistance via the miR-217/wnt3 pathway^30^. Our previous study demonstrated that HD–SB inhibited CRC progression by suppressing the expression of wnt signaling pathway genes WNT11, WNT2B, FZD2, LRP6, and PLCB4^21^. However, the potential molecular mechanism of HD–SB regulation of the wnt signaling pathway is not clear, and whether circRNAs play a regulatory role in this process deserves to be explored in depth.

In our study, we investigated the influence of HD–SB on CRC development and the mechanism of related pathways by high-throughput sequencing of circRNAs. A novel down-regulated circRNA, hsa_circ_0039933, was identified as a pro-oncogene and related to CRC proliferation, invasion, and migration, and to promote CRC progression by acting as ceRNA that regulates wnt11. This study showed that HD–SB reduced the progression of colorectal cancer cells by mediating the hsa-miR-204-5p/wnt11 by downregulating the expression of hsa_circ_0039933.

## Materials and methods

### Preparation of *Hedyotis diffusa*–*Sculellaria barbata* (HD–SB)

Take 250 g each of *Hedyotis diffusa* (HD) and *Scutellaria barbata* (SB) produced in Guilin, Guangxi, China and provided by Lingchuan Shunhong Herbal Medicine Cultivation Co., Ltd, pulverize and extract 3 times with 90% ethanol, combine the extracts, use rotary evaporator (EYELAN-2100, Japan) to concentrate into the extract. Freeze-dried into 85.15 g of extract powder, and the moisture content of extract powder was 4.1%. We declare that the collection of plant materials complies with the Wild Plants Protection Regulation of China. Permission was not necessary for collecting these species, which have not been included in the list of national key protected plants.

### Cell culture

Human colorectal cancer cell lines (HT-29, HCT-116, and SW620) were purchased from Cellcook (Guangzhou, China). HT-29 cells were cultured in L-15 high-glucose medium (CM2001, CellCook, China) with 10% fetal bovine serum (FBS, CellCook), 1% penicillin and streptomycin (P/S, 15140-122, Gibco), while HCT-116 and SW620 cells were cultured in RPMI-1640 medium (11875, Gibco) with 10% FBS, 1% P/S, then cultured in 37 °C, 5% CO_2_ incubator. SW620 cells were treated with HD–SB at 1 mg/mL, then collected after 24 h for subsequent experiments.

### High-throughput sequencing of circRNAs and bioinformatics analysis

After SW620 cells were cultured with 1 mg/mL HD–SB for 24 h, total RNA was extracted by use of TRIzol reagent (TR118-500, MRC, USA). RiboMinus™ Eukaryote System v2 kit was used to remove rRNA from total RNA, and RNase R was used to digest linear RNA to enrich circRNAs. RNA was fragmented for circRNAs library construction using the TrueLib mRNA Library Prep Kit for Illumina. The RNA was reverse transcribed to synthesize a two-stranded product, repaired at its end with a plus dA tail, and ligated to Adapter. The enriched product was amplified by PCR reaction. After that, the fragments were screened and purified to obtain the desired libraries. Finally, the constructed libraries were tested for quality and yield using an Agilent 2100 Bioanalyzer and a Qubit 3.0 fluorometer, and the libraries were sequenced using an Illumina HiSeq2500 platform (Illumina, USA).

### Bioinformatics analysis

Quality control of the raw reads by Illumina HiSeqTM2500 sequencing was assessed by Fast-QC (http://www.bioinformatics.babraham.ac.uk/projects/fastqc/). The remaining clean reads were mapped to the reference human genome (GRCh38) using HISAT2 software (version 2.1.0, https://daehwankimlab.github.io/hisat2/). To predict circRNAs, circRNAs were identified and quantified from mapped splice junction reads using ACFS2 (https://github.com/arthuryxt/acfs). Differentially expressed circRNAs between HD–SB group and control were analyzed using the DE-Seq algorithm, with significant differences defined as absolute |log2 fold-change|> 1 and p value < 0.05. The differentially expressed circRNAs and their functional enrichment were further analyzed by Gene Ontology (GO) and KEGG database^31–33^ in DAVID software (6.8). hsa_circ_0039933-miRNAs-wnt11 networks were predicted by miRanda 3.3 and drawn by cytoscape 3.2.1.

### Quantitative real-time PCR

Total RNA was obtained from cells using TRIzol reagent, and RNA was reverse transcribed into cDNA by reverse transcription kit (Takara, Japan). Relative gene expression was detected by real-time PCR on cDNA products using SYBR Green Premix kit (Takara, Japan). GAPDH was the internal reference gene of circRNA/mRNA, and U6 was used as the internal reference of miRNA. The relative gene level was calculated by the 2^−ΔΔCt^ method and the primer sequences are shown in Table 1.

**Table 1:** The sequences of the primer.

The primer	The sequences
hsa-miR-1225-3p-RT	GTCGTATCCAGTGCAGGGTCCGAGGTATTCGCACTGGATACGACCTGGGG
hsa-miR-1225-3p-F	TGAGCCCCTGTGCCGCC
hsa-miR-1299-RT	GTCGTATCCAGTGCAGGGTCCGAGGTATTCGCACTGGATACGACTCCCTC
hsa-miR-1299-F	TTCTGGAATTCTGTGTGA
hsa-miR-4469-RT	GTCGTATCCAGTGCAGGGTCCGAGGTATTCGCACTGGATACGACTCCGAG
hsa-miR-4469-F	GCTCCCTCTAGGGTCGCT
hsa-miR-873-3p-RT	GTCGTATCCAGTGCAGGGTCCGAGGTATTCGCACTGGATACGACTCCCGG
hsa-miR-873-3p-F	GGAGACTGATGAGTTCC
hsa-miR-766-5p RT	GTCGTATCCAGTGCAGGGTCCGAGGTATTCGCACTGGATACGACAAGACC
hsa-miR-766-5p F	AGGAGGAATTGGTGCT
hsa-miR-204-5p RT	GTCGTATCCAGTGCAGGGTCCGAGGTATTCGCACTGGATACGACAGGCAT
hsa-miR-204-5p F	TTCCCTTTGTCATCCT
U6-F	CTCGCTTCGGCAGCACA
U6-R	AACGCTTCACGAATTTGCGT
H-WNT11-F	ATTTGCTTGACCTGGAGAGAGG
H-WNT11-R	TGAGGTTGTCCGCACATCC
hsa_circ_0004083-CF1	TCCGATTCCCATCCCACTTC
hsa_circ_0004083-CR1	AAACTCTTTCACTTCCTCTGGT
hsa_circ_0004145-CF1	CCTGACACTGTAACACTATGCC
hsa_circ_0004145-CR1	GTGCACCACTGAATGACCTT
hsa_circ_0007580-CF1	TTATATCGCCCCAGAGAAAGC
hsa_circ_0007580-CR1	TTCTTCTGTGCCCTTCCTGT
hsa_circ_0023214-CF1	GCATTGAGAGCTGTGACCTG
hsa_circ_0023214-CR1	GAAGGTGGTGGGCGAGAA
hsa_circ_0039933-CF1	AAGGGGCTCACATTGTCCTT
hsa_circ_0039933-CR1	GGAAGTTCTTGAGCAGACCG
hsa_circ_0067434-CF1	CTCCAGAGACTTGTTCCCCA
hsa_circ_0067434-CR1	AGACCTCGCAGCTTACAACT
hsa_circ_0023214-LF	ACGACCTCAGCATCGACATC
hsa_circ_0023214-LR	AGTACAGGTACCCTCGCTCC
hsa_circ_0039933-LF	CCAGCGGTCTGCTCAAGAAC
hsa_circ_0039933-LR	CAAGGACAATGTGAGCCCCT
hsa_circ_0039933-LF	CCAGCGGTCTGCTCAAGAAC
hsa_circ_0039933-LR	CAAGGACAATGTGAGCCCCT
H-GAPDH-F	GAGTCAACGGATTTGGTCGT
H-GAPDH-R	GACAAGCTTCCCGTTCTCAG

### Cell transfection

The OV-hsa_circ_0039933 overexpression plasmid was synthesized in General Biologicals (Anhui, China). The small interfering RNA (siRNA) interference fragments of hsa_circ_0039933, hsa-miR-204-5p mimics and inhibitors were devised and synthesized by Gene Pharma Company (Shanghai, China). According to the manufacturer's instructions, cells were transfected with 200 pmol of siRNA or 2.5 μg overexpression plasmids using Lipofectamine 3000 reagent (Invitrogen, USA). Sequences for inhibitors, mimics and siRNAs are given in Table 2.

**Table 2:** The sequences for inhibitor, mimics and siRNAs.

Name	The sequences
Hsa_circ_0039933-siRNA1	Sense: UACACCAGCCCAGCGGUCUTT Antisense: AGACCGCUGGGCUGGUGUAUA
Hsa_circ_0039933-siRNA2	Sense: UACUUAUACACCAGCCCAGTT Antisense: CUGGGCUGGUGUAUAAGUAAA
Hsa_circ_0039933-siRNA3	Sense: UACACCAGCCCAGCGGUCUTT Antisense: AGCAGACCGCUGGGCUGGUGU
Hsa_circ_0039933-siNC	Sense: UUCUUCGAACGUGUCACGUTT Antisense: ACGUGACACGUUCGGAGAATT
Hsa-miR-204-5p mimics	Sense: UUCCCUUUGUCAUCCUAUGCCU Antisense: GCAUAGGAUGACAAAGGGAAUU
Hsa-miR-204-5p NC mimics	Sense: UUCUCCGAACGUGUCACGUTT Antisense: ACGUGACACGUUCGGAGAATT
Hsa-miR-204-5p inhibitor	AGGCAUAGGAUGACAAAGGGAA
Hsa-miR-204-5p NC inhibitor	CAGUACUUUUGUGUAGUACAA

### Cell viability

When the cells were in a logarithmic growth phase, they were digested with 0.25% trypsin to form a single-cell suspension. Then cells were collected and seeded into 96-well plates at a concentration of 5 × 10^3^ cells/mL. When the cells adhered, CCK8 solution (Vazyme, China) was added to each well, 10 μL of detection reagent was added to 100 μL of medium and incubated in an incubator for 2–4 h. Then the absorbance at 450 nm was detected by a microplate reader.

### Luciferase reporter assay

The hsa_circ_0039933 sequence containing potential wild-type or mutant binding sites was constructed into the pmirGLO dual-luciferase vector (Promega Corporation, Fitchburg, WI, USA), pmirGLO-hsa_circ_0039933 wild-type or mutant and hsa-miR-204-5p mimics or negative control were co-transfected with Lipofectamine 3000 (Invitrogen, USA). Luciferase activity was detected using a dual-luciferase reporter kit (Promega Corporation, USA) after 48 h of transfection.

### Cell apoptosis

Cell apoptosis was performed by Annexin V-APC/7-AAD double staining apoptosis detection kit (KGA1026, KeyGen, China). The cells in the 6-well plate were made into cell suspension with 0.25% trypsin, washed three times with PBS, and centrifuged at 1000 rpm for 5 min to remove the PBS. Next, resuspend the cells in 200 μL buffer solution, and added 5 μL Annexin V-APC and 5 μL 7-AAD solution. The cells were incubated for 10 min at room temperature. The total apoptotic cells were early-apoptotic cells and late-apoptotic cells.

### Transwell assay

Cells were seeded in transwell chambers containing serum-free medium at 2 × 10^4^ cells/mL, and medium containing 10% FBS was placed in the lower chamber. After 48 h of culture, the migrated cells were fixed with 4% paraformaldehyde followed by crystal violet staining. The cells in five separate areas were measured and averaged. For cell invasion assay, the upper chamber was precoated with Matrigel (BD Biosciences, China). 2 × 10^4^ cells were added to the Matrigel invasion chamber, and medium containing 10% FBS was placed in the lower chamber. After 96 h of culture, non-invasive cells were discarded, and invasive cells were fixed and stained with crystal violet.

### Western blot assay

High performance cell lysate (P0013B, Beyotime, China) was added to the cells, then lysed for 20 min until the cells were completely ruptured. The supernatant protein was carefully aspirated after centrifugation at 13,300 rpm for 15 min. The proteins were separated by electrophoresis on 10% SDS-PAGE gel and transferred to PVDF membranes (Invitrogen, USA). After blocking with 5% bovine serum albumin (BSA) for 1 h, the cells were incubated with primary antibody overnight at 4 °C. Then it was incubated with HRP-labeled secondary antibody (Jackson) for 1 h, and finally the bands were detected by chemiluminescence. Information for primary antibodies: anti-wnt11 (ab31962, Abcam), anti-β-catenin (ab32572, Abcam), anti-GAPDH (60004-1-lg, Proteintech).

### Statistical analysis

GraphPad Prism 8.0 was used for statistical analysis, and experimental data were presented as mean ± standard deviation (SD). Each experiment had at least three replicates. Differences between groups were analyzed using Student's t-test or one-way/two-way ANOVA. p-Value < 0.05 was considered statistically significant.

## Results

### High-throughput sequencing analysis of circRNAs in colorectal cancer cells

**Figure 1:**
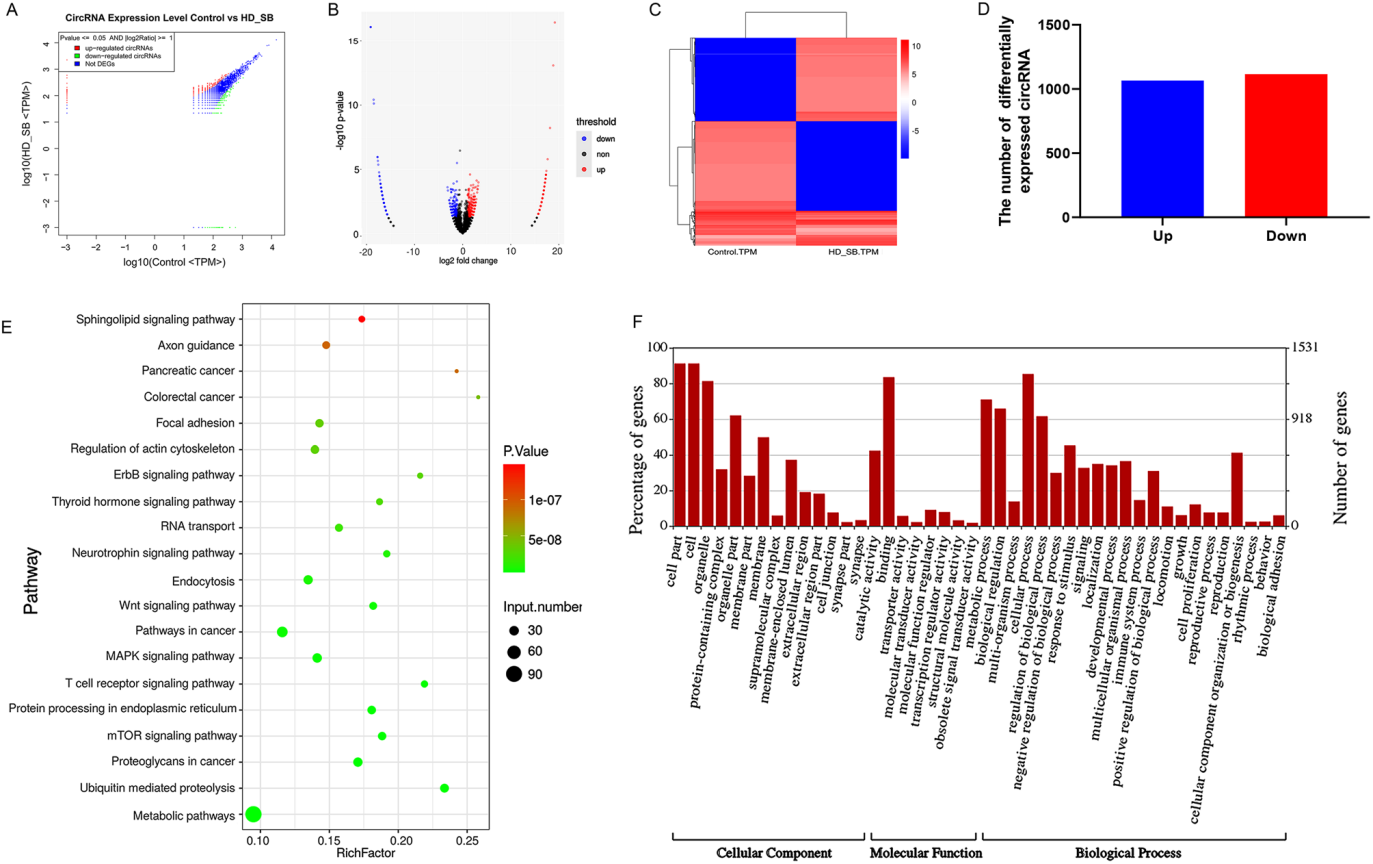
Bioinformatic analysis of circRNAs transcriptome sequencing. (**A**) Scatter plot of differentially expressed circRNAs in control and HD–SB groups. (**B**) Volcano plot of differentially expressed circRNAs in control and HD–SB groups. (**C**) Heatmap of differentially expressed circRNAs in control and HD–SB groups. Heatmap colors reflect relative abundance from low (blue) to high (red). (**D**) The number of differentially expressed circRNAs in the control and HD–SB groups. (**E**) Signaling pathway (KEGG) analyses of the differentially expressed circRNAs in the control and HD–SB groups. (**F**) Gene Ontology (GO) enrichment analysis of the differentially expressed circRNAs in the control and HD–SB groups.

Here, we performed high-throughput sequencing of circRNAs after treating colorectal cancer cells (SW620) with HD–SB at 1 mg/mL. Volcano and scatter plots exhibited changes in circRNAs expression between control and HD–SB treated groups (Fig. 1A,B). A total of 2181 circRNAs were differentially expressed in the HD–SB group by heat map analysis, of which 1066 were up-regulated and 1115 were down-regulated (Fig. 1C,D). Then differentially expressed circRNAs were analyzed by use of KEGG and GO enrichment analysis. KEGG analysis showed that the Wnt signaling pathway and sphingolipid signaling pathway were enriched, while GO analysis showed that differential circRNAs were mainly enriched in cellular progress and cell part (Fig. 1E,F).

### Hsa_circ_0039933 was downregulated in colorectal cancer cells

**Figure 2:**
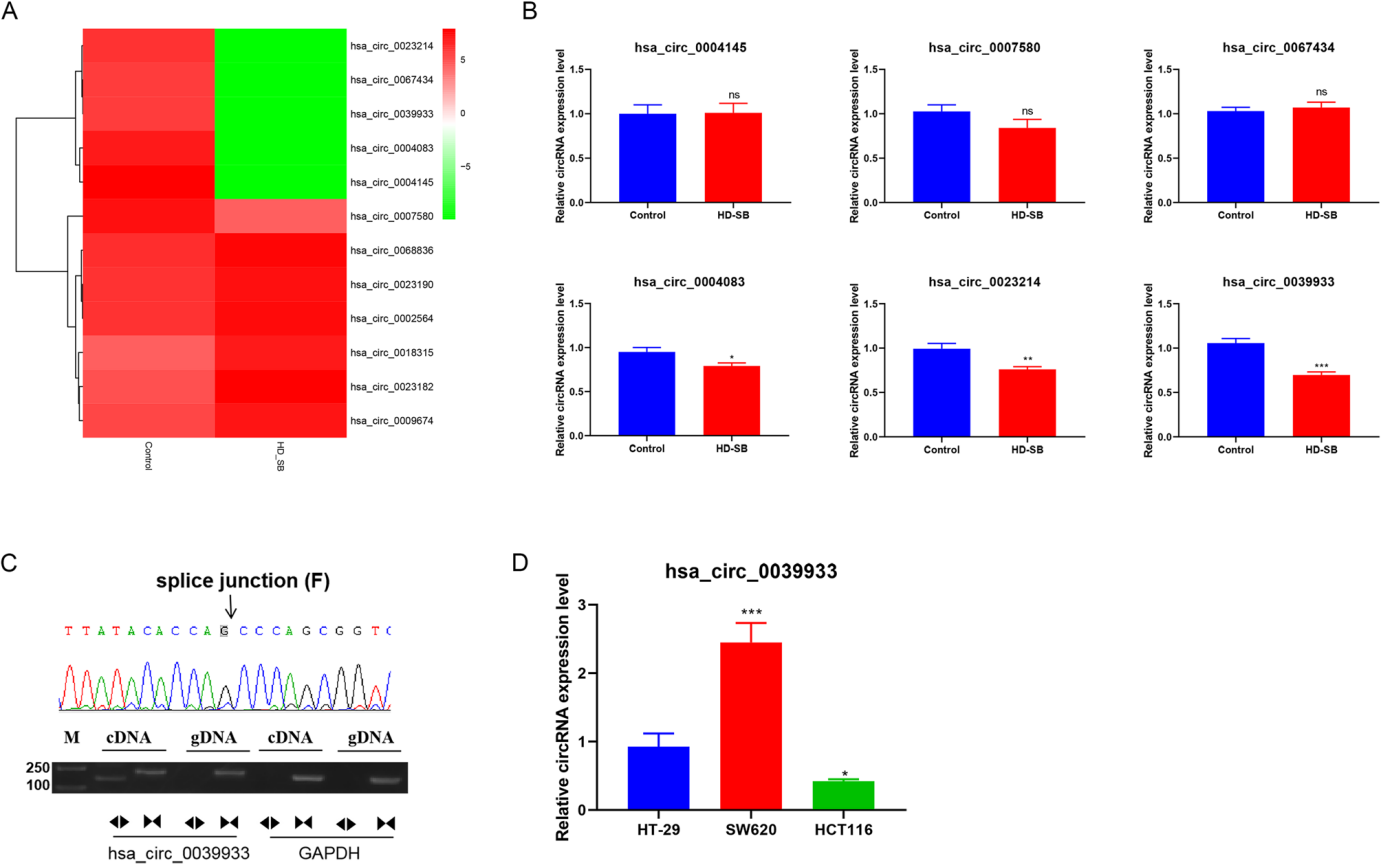
Hsa_circ_0039933 was downregulated in colorectal cancer cells after treatment with HD–SB. (**A**) Differential circRNAs expression profiles targeting the WNT pathway. (**B**) qPCR detection of the expression of 6 circRNAs down-regulated after HD–SB treatment. (**C**) Sanger sequencing proved the splice junction (arrow) of hsa_circ_0039933. (**D**) The level of hsa_circ_0039933 in 3 colorectal cancer cells (HT-29, SW620, and HCT116) was detected by qPCR. Asterisk represents *p*-values, vs HT-29, *p < 0.05, **p < 0.01, ***p < 0.001.

Our previous study demonstrated that the Wnt pathway was suppressed in HD-SB-treated colorectal cancer cells, and the down-regulation of wnt11 was the most obvious^21^. Therefore, we predicted differential circRNAs that might target regulation of wnt11 based on ceRNA mechanisms (Fig. 2A). We further validated the expression of circRNAs consistent with wnt11, the results showed that hsa_circ_0039933 was apparently reduced in HD-SB treatment of SW620 cells (Fig. 2B). Sanger sequencing further confirmed the existence of hsa_circ_0039933 cyclization site (Fig. 2C), so hsa_circ_0039933 was selected for subsequent experimental studies. Then the expression of hsa_circ_0039933 was confirmed in three colorectal cancer cell lines (HT-29, SW620, and HCT116 cells), and it was proved that hsa_circ_0039933 had the highest expression in SW620 cells and the lowest expression in HCT116 cells (Fig. 2D). Follow-up functional studies were carried out using these two cells.

### Hsa_circ_0039933 regulated colorectal cancer cell proliferation and migration ability

**Figure 3:**
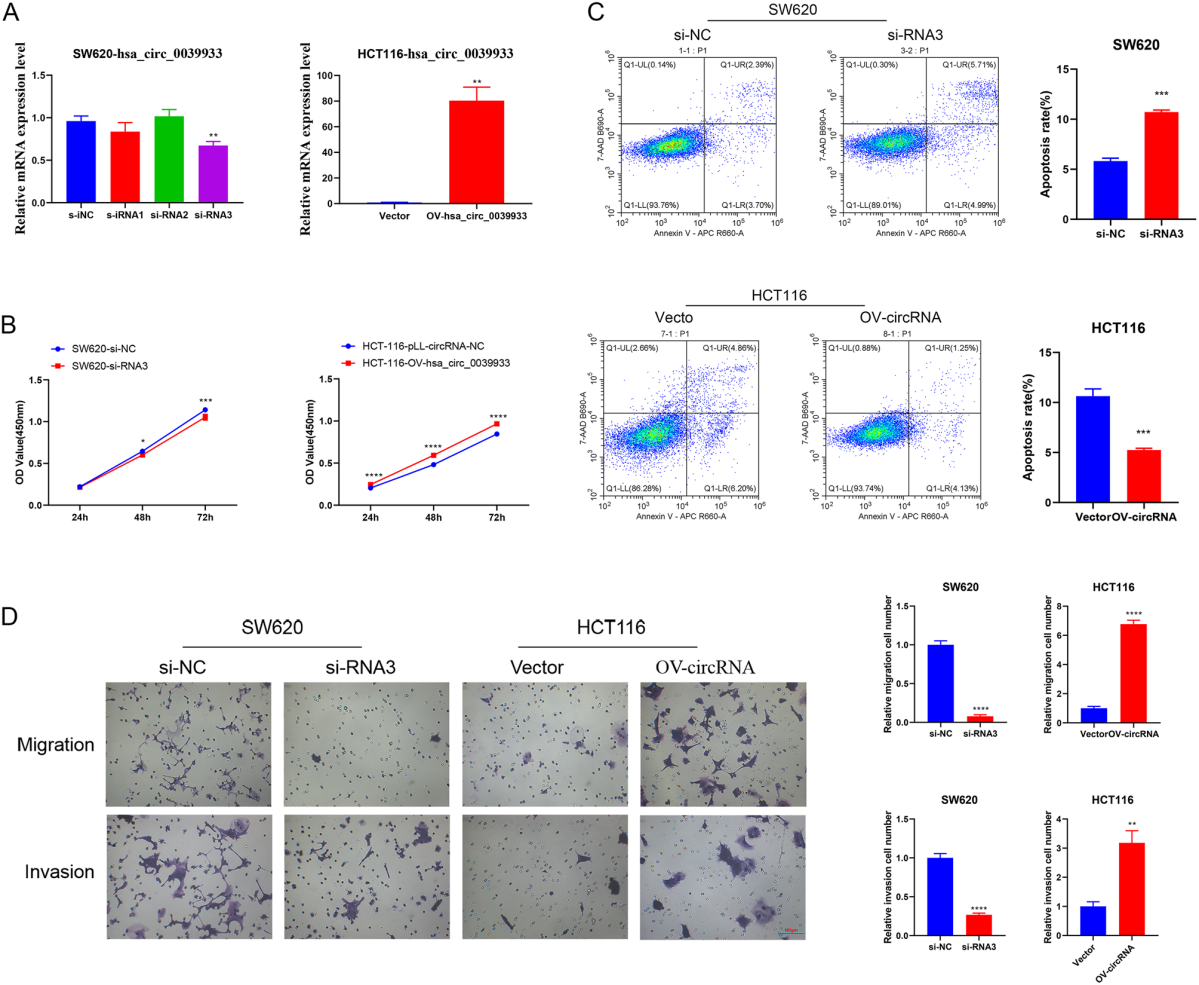
The effects of hsa_circ_0039933 on the function of colorectal cancer cells. The hsa_circ_0039933 small interference RNA fragment (si-RNA) was transfected in SW620 cells and the overexpressing hsa_circ_0039933 plasmid (OV-circRNA) was constructed in HCT116 cells, respectively. (**A**) The interference effect of si-RNAs in SW620 cells and the overexpression effect of OV-circRNA in HCT116 cells were detected by qPCR. (**B**) The effect of siRNA on the proliferation of SW620 cells and the effect of OV-circRNA on the proliferation of HCT116 cells were detected by CCK8 assay. (**C**) The apoptosis effect of siRNA on SW620 cells and OV-circRNA on HCT116 cells were measured by use of flow cytometry. (**D**) Transwell experiments were used to detect the migration and invasion changes of SW620 cells transfected by siRNA and HCT116 cells transfected by OV-circRNA. Asterisk represents *p*-values, *p < 0.05, **p < 0.01, ***p < 0.001, ****p < 0.0001.

To explore the effect of hsa_circ_0039933 in colorectal cancer cells. We chose SW620 cells with the highest hsa_circ_0039933 expression for post-interference functional studies, while HCT116 with the lowest hsa_circ_0039933 expression was chosen for post-overexpression functional studies. The interference effect of si-RNA3 was the most obvious, and the overexpression plasmid was successfully constructed via qPCR assay (Fig. 3A). Through the analysis of cell viability, after interfering with the expression of hsa_circ_0039933, the proliferation ability of SW620 cells was weakened, while the proliferation ability of HCT116 cells was enhanced after overexpression of hsa_circ_0039933 (Fig. 3B). Flow detection of cell apoptosis revealed that after interfering with hsa_circ_0039933, the apoptosis of SW620 cells increased, while the number of apoptotic cells in HCT116 decreased after overexpression of hsa_circ_0039933 (Fig. 3C). In addition, interfering with the expression of hsa_circ_0039933 inhibited the migration and invasion of SW620 cells, whereas overexpression of hsa_circ_0039933 did the opposite in HCT116 cells (Fig. 3D).

### Hsa_circ_0039933 sponges hsa-miR-204-5p

**Figure 4:**
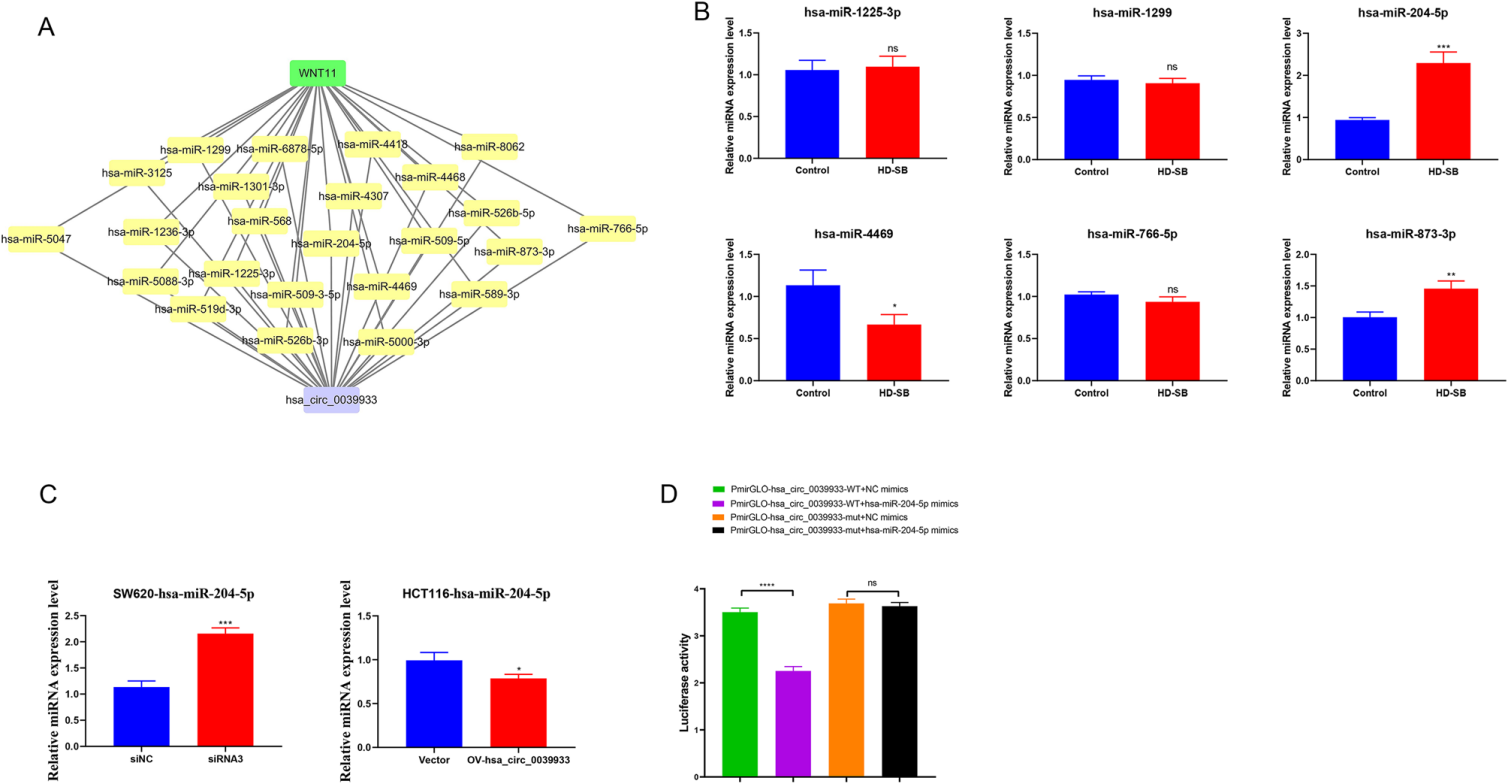
Hsa_circ_0039933 sponges hsa-miR-204-5p. (**A**) The interaction network diagram of hsa_circ_0039933-miRNA-wnt11 predicted by Miranda software. (**B**) The expression of 6 miRNAs was detected by qPCR. (**C**) The expression of hsa-miR-204-5p was examined by qPCR in SW620 cells with interference of hsa_circ_0039933 and HCT116 cells with overexpression of hsa_circ_0039933, respectively. (**D**) Dual luciferase reporter assay validated the association between hsa_circ_0039933 and hsa-miR-204-5p. Asterisk represents *p*-values, *p < 0.05, **p < 0.01, ***p < 0.001, ****p < 0.0001.

The hsa_circ_0039933-miRNA-wnt11 interaction network map of the Wnt pathway was predicted by Miranda software, and there were 24 miRNAs targeting wnt11 (Fig. 4A). We validated 6 miRNAs with multi-binding sites and associated with cancer, among which the levels of hsa-miR-873-3p and hsa-miR-204-5p increased, which was consistent with the ceRNA theory, and the level of hsa-miR-204-5p elevated more significantly, as a follow-up research molecule (Fig. 4B). Further, we detected the hsa-miR-204-5p in two cell lines with knockdown and overexpression of hsa_circ_0039933, respectively. The results demonstrated that the expression of hsa-miR-204-5p was increased in SW620 cells with knockdown of hsa_circ_0039933, while decreased in HCT116 cells with overexpression hsa_circ_0039933 (Fig. 4C). To investigate the binding relationship between hsa_circ_0039933 and hsa-miR-204-5p, we co-transfected hsa_circ_0039933-WT and hsa-miR-204-5p mimics in 293 T cells, the dual luciferase activity decreased obviously compared to that of the cotransfected hsa_circ_0039933-WT and NC mimics. However, co-transfection of hsa_circ_0039933-mut and hsa-miR-204-5p mimics had no obvious effect compared to that of the cotransfected hsa_circ_0039933-WT and NC mimics, indicating that hsa_circ_0039933 and hsa-miR-204-5p have binding sites (Fig. 4D).

### Hsa_circ_0039933 regulated colorectal cancer proliferation via hsa-miR-204-5p/wnt11

**Figure 5:**
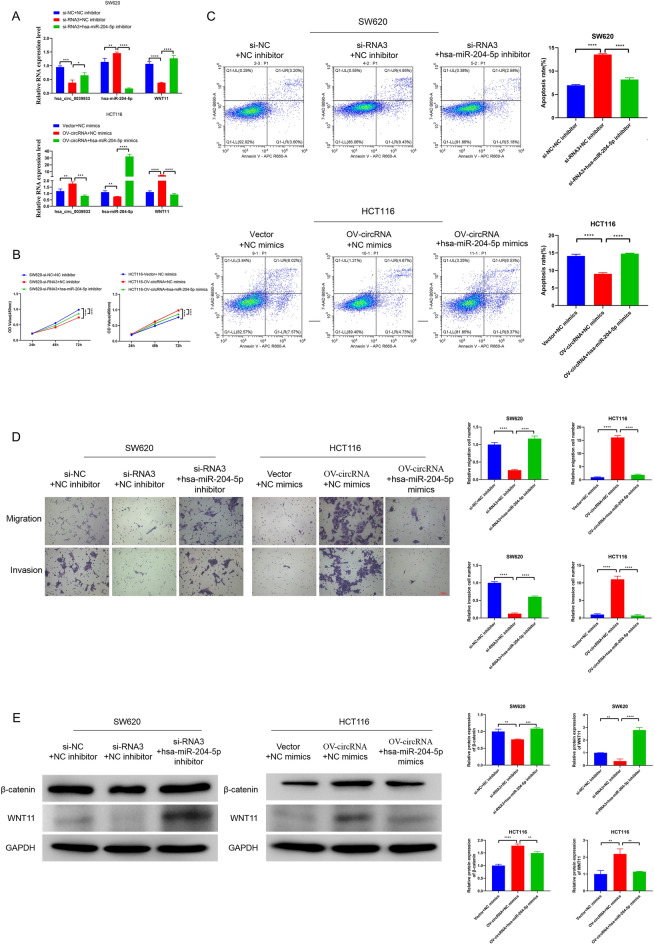
Hsa_circ_0039933 regulated colorectal cancer proliferation via hsa-miR-204-5p/wnt11. After co-transfection of hsa_circ_0039933 si-RNA3 and hsa-miR-204-5p inhibitor in SW620 cells, and co-transfection of OV-hsa_circ_0039933 and hsa-miR-204-5p mimics in HCT116 cells for 48 h. (**A**) The expression of hsa_circ_0039933, hsa-miR-204- 5p and wnt11 was detected by qPCR. (**B**) Cell viability of SW620 and HCT-116 cells was determined by CCK8. (**C**) Apoptosis of SW620 and HCT-116 cells was counted by flow cytometry. (**D**) The migration and invasion abilities of SW620 and HCT-116 cells were detected by Transwell assays. (**E**) The expression of Wnt pathway proteins (β-catenin and wnt11) in SW620 and HCT116 cells was assessed by western blot experiments. All the blots were cut before hybridization with the antibodies. Asterisk represents *p*-values, *p < 0.05, **p < 0.01, ***p < 0.001, ****p < 0.0001.

In order to clarify the regulatory influence of hsa_circ_0039933 and hsa-miR-204-5p on colorectal cancer cells. We co-transfected hsa_circ_0039933 si-RNA3 and hsa-miR-204-5p inhibitor in SW620 cells, and co-transfected OV-hsa_circ_0039933 and hsa-miR-204-5p mimics in HCT116 cells, respectively. It was revealed that hsa-miR-204-5p inhibitor could restore the inhibitory effect of hsa_circ_0039933 si-RNA3 on wnt11, while hsa-miR-204-5p mimics could attenuate the promotion of OV-hsa_circ_0039933 on wnt11 (Fig. 5A). Moreover, hsa-miR-204-5p inhibitor restored the inhibitory effect of hsa_circ_0039933 si-RNA3 on the proliferation, migration and invasion of SW620 cells, while hsa-miR-204-5p mimics reversed the promotion effects of OV-hsa_circ_0039933 on the proliferation, migration, and invasion of HCT116 cells (Fig. 5B–D). We detected the Wnt pathway-related proteins wnt11 and β-catenin, hsa_circ_0039933 si-RNA3 inhibited the expression of wnt11 and β-catenin, but was restored by inhibiting hsa-miR-204-5p, while OV-hsa_circ_0039933 promoted wnt11 and β-catenin, but was suppressed by hsa-miR-204-5p mimics (Fig. 5E), suggesting that hsa_circ_0039933 played a crucial role in indirectly regulating wnt11 by sponging hsa-miR-204-5p.

## Discussion

Heat-clearing and detoxifying herbs play a pivotal role in the treatment of CRC in TCM, among them, HD and SB are the popular heat clearing and detoxifying TCM used in cancer treatment and are the customary TCM pair for the treatment of breast and bladder cancer^20,34^. HD contains terpenoids, anthraquinones and other compounds that have anti-tumor effects and can inhibit the development of many tumors^35^. Previous studies have reported that HD inhibited CRC growth by inducing apoptosis and inhibiting tumor angiogenesis^36^. In addition, SB is a genus of Scutellaria in the family Labiatae, containing a variety of active ingredients with anti-tumor activity, and has been reported to have inhibitory effects in a variety of malignant tumors, such as SB significantly inhibited TGF-β1-induced CRC migration and invasion^37^. Based on the properties of both, there is a growing tendency for studies to use a combination of HD and SB. HD–SB reduced the phosphorylation levels of ERK, NF-κB, JNK, and p38 MAPK, which attenuated lung cancer cell proliferation and colony formation^38^. HD–SB weakened anti-apoptotic proteins Bcl-2 and Mcl-1, and mediated the activation of apoptotic protein caspase-3 in bladder cancer cells^20^. Our previous study demonstrated that HD, SB, and HD–SB showed a significant decrease in cell viability after intervention in colorectal cancer HCT-29 and SW620 cells^21^. And the combination of HD and SB was more effective in regulating the development of CRC.

Differential expression profiles of circRNAs are the basis for identifying novel tumor oncogenic/anti-tumor factors and elucidating the underlying mechanisms of their biological functions^39^. Disciplinary research to identify circRNAs as regulators and valuable disease diagnostic markers is increasing. We investigated the potential role of circRNAs in HD-SB, including 1066 upregulated circRNAs and 1115 downregulated circRNAs, and identified hsa_circ_0039933 as a tumor-promoting factor in CRC. In our study, differentially expressed circRNAs in HD–SB group were mostly enriched in metabolism-related signaling pathways, cancer-related pathways, MAPK signaling pathways, and Wnt signaling pathways. Most circRNAs are rich in miRNA response elements, which bind to them by competing with endogenous RNAs, thereby inhibiting miRNA function and regulating gene expression^24,25^. Studies have shown that circ-001971 as a ceRNA attenuated miR-29c-3p-induced VEGFA inhibition and affected endothelial cell angiogenesis through the tumor microenvironment, thereby aggravating CRC proliferation, invasion, and angiogenesis^40^. CircEIF3K regulated CRC tumorigenesis and metastasis via miR-214/PD-L1, and knockdown of circEIF3K inhibited tumor growth^41^. Shang et al. identified that the exosome circPACRGL played a carcinogenic role in CRC metastasis, enhanced cell migration, invasion and promoted the differentiation of N1-N2 neutrophils through the miR-142-3p/miR-506-3p axis^42^. We predicted that hsa-miR-204-5p was the ceRNA of hsa_circ_0039933 by Miranda software. Overexpression of hsa_circ_0039933 promoted the proliferation, invasion, and migration of colorectal cancer cells, which could be reversed after upregulation of hsa-miR-204-5p. In addition, it was predicted that the target gene of hsa-miR-204-5p was wnt11.

Wnt signaling is an indispensable cellular process in cell development and differentiation, but its aberrant activation leads to the progress of various cancers, including CRC^43,44^. Activated Wnt pathway plays a vital function in CRC progression and metastasis, in which cell surface ligands are initiated by Wnt stimulation, resulting in β-catenin activation and upregulation of Wnt-responsive genes^45^. At present, at least 19 Wnt ligands have been identified in CRC tissues^46^. It was reported that the expression of wnt11 was apparently up-regulated in CRC tumors, and its content was positively correlated with poor CRC prognosis, which made wnt11 a potential biomarker and therapeutic target in CRC^47^. Consistent with this, we previously found that after HD-SB treatment of colorectal cancer cells, the levels of wnt11, wnt2B, FZD2, LRP6, and PLCB45 were down-regulated, among which wnt11 was the most significantly down-regulated^21^. High expression of lncABHD11-AS1 was associated with poor overall survival in CRC patients, and it acts as a molecular sponge for miR-1254 targeting wnt11 to accelerate CRC cell proliferation, invasion and tumor growth^48^. In order to explore the molecular mechanism of HD-SB down-regulation of wnt11 protein, hsa-miR-204-5p was identified as a regulatory gene of wnt11 in this study. We demonstrated that inhibiting the expression of hsa-miR-204-5p could reverse the down-regulation of wnt11, cell apoptosis, proliferation and migration ability caused by interfering with hsa_circ_0039933, indicating that HD-SB inhibited the expression of wnt11 through hsa_circ_0039933/hsa-miR-204-5p and exerted anti-tumor effect. Despite the lack of validation at the animal level and trial in clinical studies, our study adds credibility to the application of HD-SB by revealing its efficacy in treating cancer at the molecular level of circRNAs, and after further validation, we believe that HD-SB is expected to be a clinically useful herbal medicine for CRC.

## Conclusion

In general, we explored the molecular mechanism of HD-SB in the treatment of CRC based on transcriptional sequencing. It was demonstrated that HD-SB down-regulated the expression of wnt11 through the hsa_circ_0039933/hsa-miR-204-5p axis, thereby inhibiting the Wnt signaling pathway and reducing the proliferation, invasion, and migration ability of colorectal cancer cells. This study revealed the underlying mechanism of HD-SB exerting its antitumor effect and confirmed that hsa_circ_0039933/hsa-miR-204-5p/wnt11 could be important therapeutic targets for CRC.

### Supplementary Information

Supplementary Information 1.

Supplementary Information 2.

## Data Availability

The dataset generated and analysed during the current study is available in the Gene Expression Omnibus (GEO) repository, GSE237129 (enter secure: stavcuyyvfwptah).
