# Supplementary material for: Hedyotis diffusa–Sculellaria barbata (HD–SB) suppresses the progression of colorectal cancer cells via the hsa_circ_0039933/hsa-miR-204-5p/wnt11 axis
Source: Sci Rep. 2023 Aug 16;13:13331. doi: 10.1038/s41598-023-40393-1 (PMC10432535; doi:10.1038/s41598-023-40393-1)
Supplement: Supplementary file 1 — Supplementary Information 1. [file 41598_2023_40393_MOESM1_ESM.pdf]

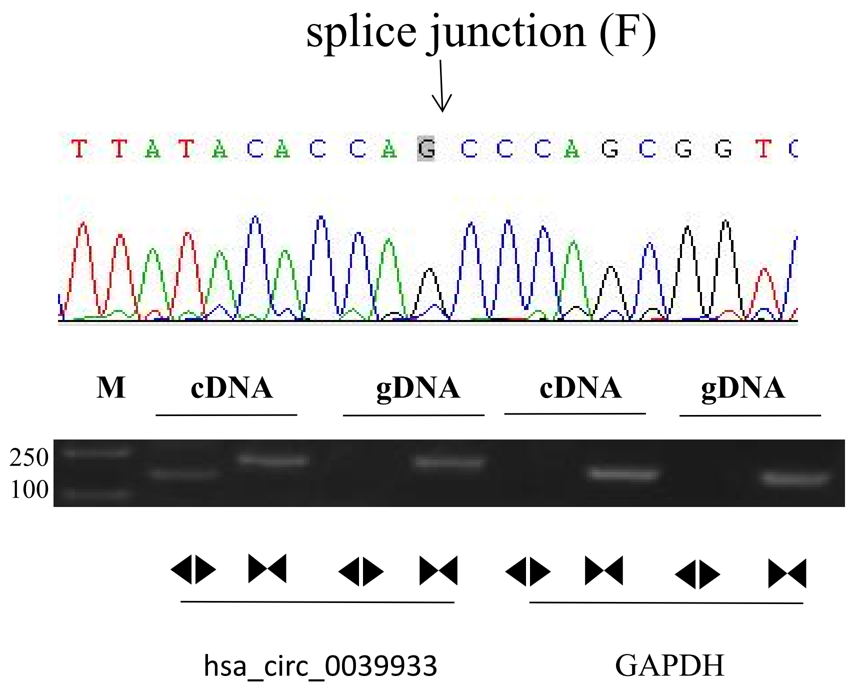

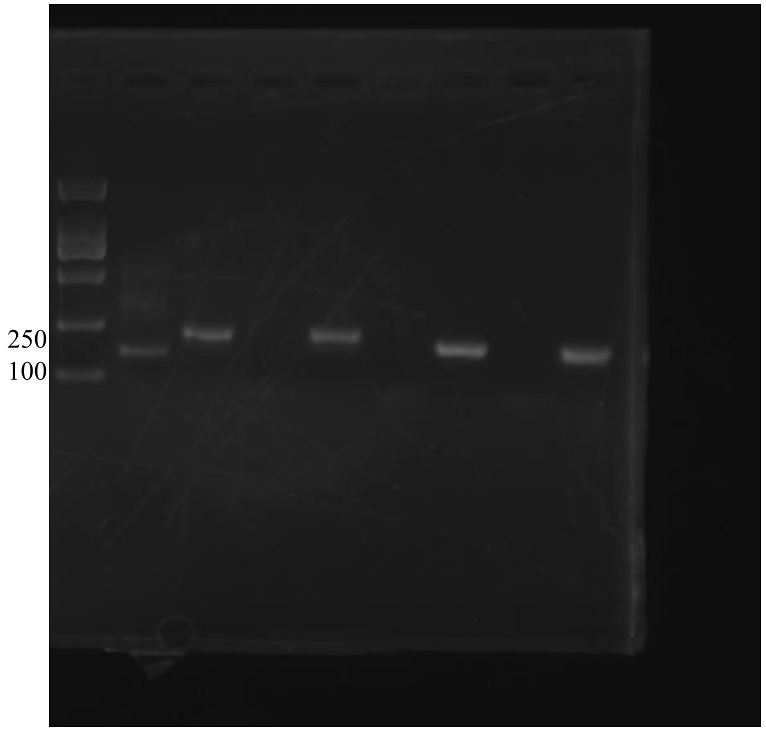

The left half of this gel was cropped because the left side was the results of other subjects that were not conveniently presented here.
