# Supplementary material for: Hedyotis diffusa–Sculellaria barbata (HD–SB) suppresses the progression of colorectal cancer cells via the hsa_circ_0039933/hsa-miR-204-5p/wnt11 axis
Source: Sci Rep. 2023 Aug 16;13:13331. doi: 10.1038/s41598-023-40393-1 (PMC10432535; doi:10.1038/s41598-023-40393-1)
Supplement: Supplementary file 2 — Supplementary Information 2. [file 41598_2023_40393_MOESM2_ESM.pdf]

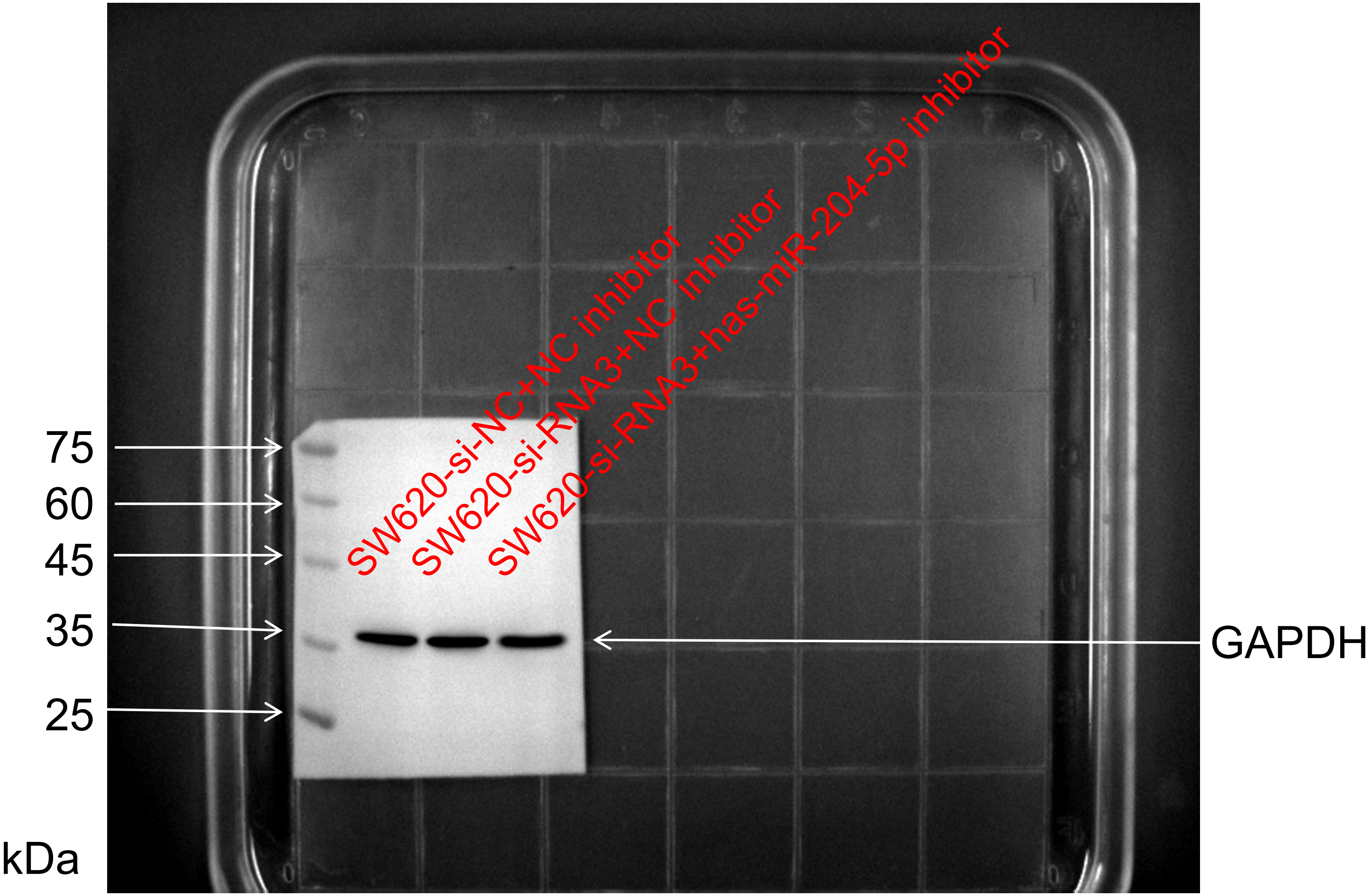

GAPDH, Proteintech, 60004-1-Ig, 1:10000, 36kD;
anti-Mouse IgG, Jackson, 115-035-003, 1:5000

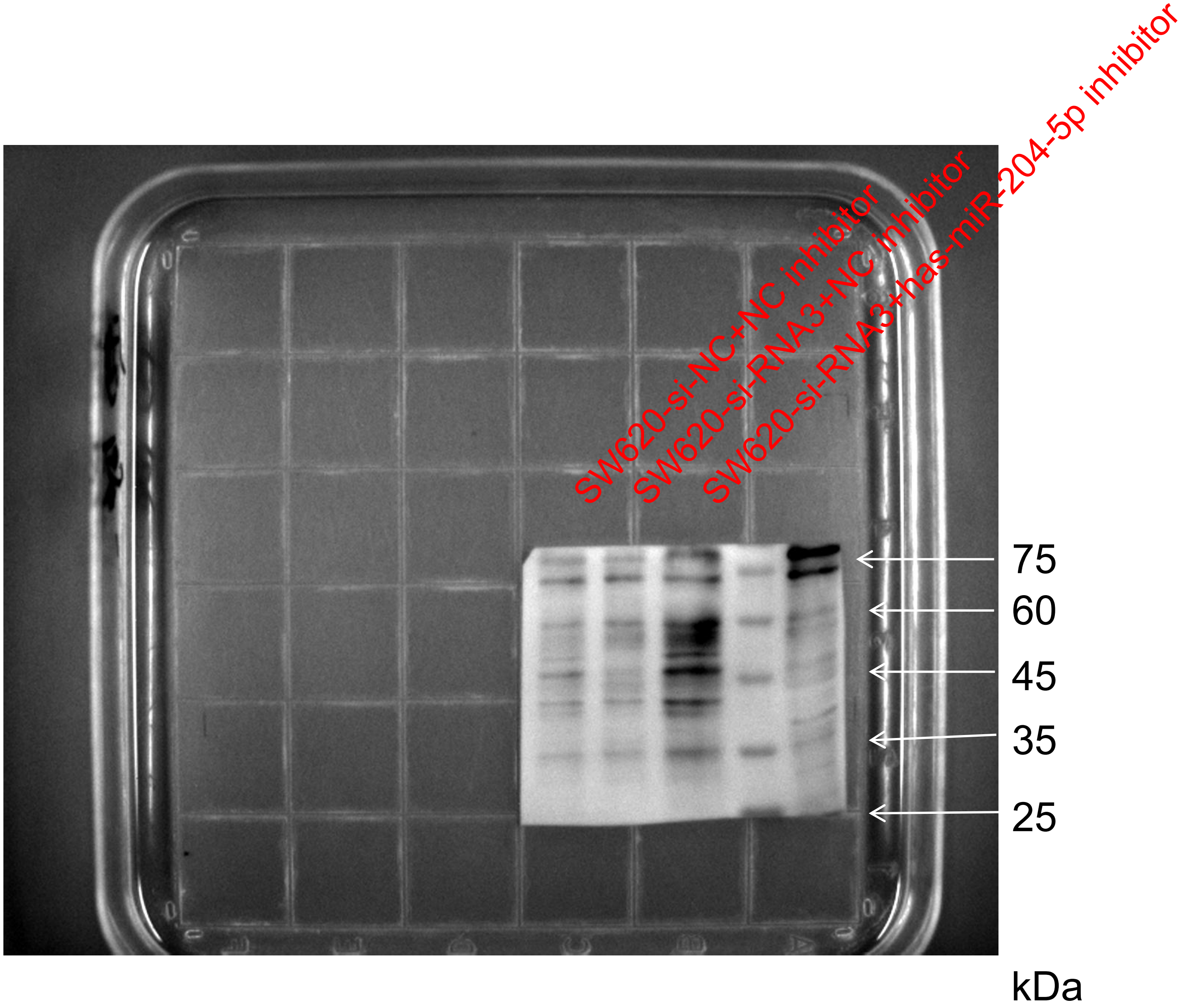

WNT11, Abcam, ab31962, 1:1000, 39kD;
anti-Rabbit IgG, Jackson, 111-035-003, 1:2000

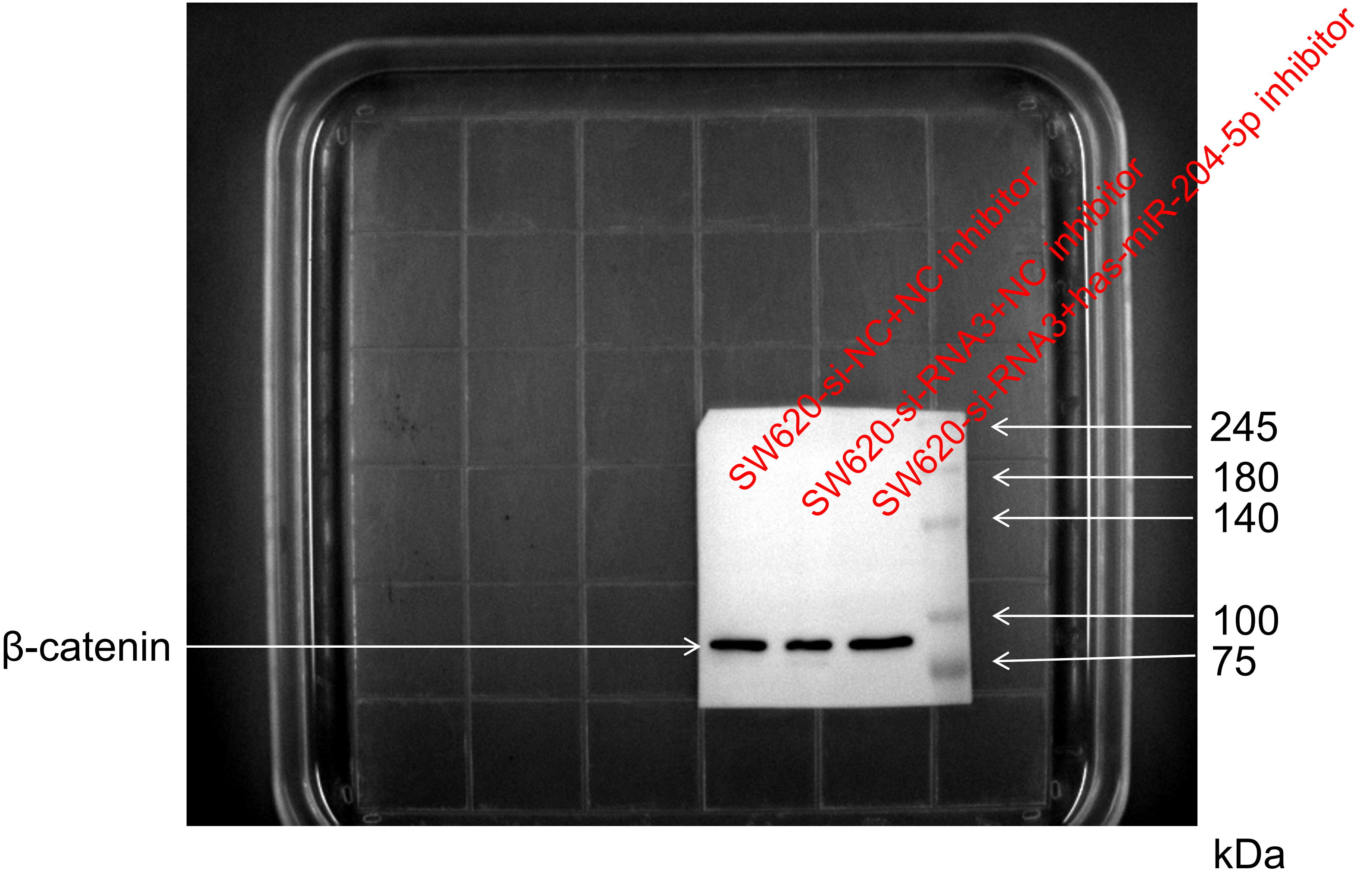

β-catenin, Abcam, ab32572, 1:1000, 92kD;
anti-Rabbit IgG, Jackson, 111-035-003, 1:2000

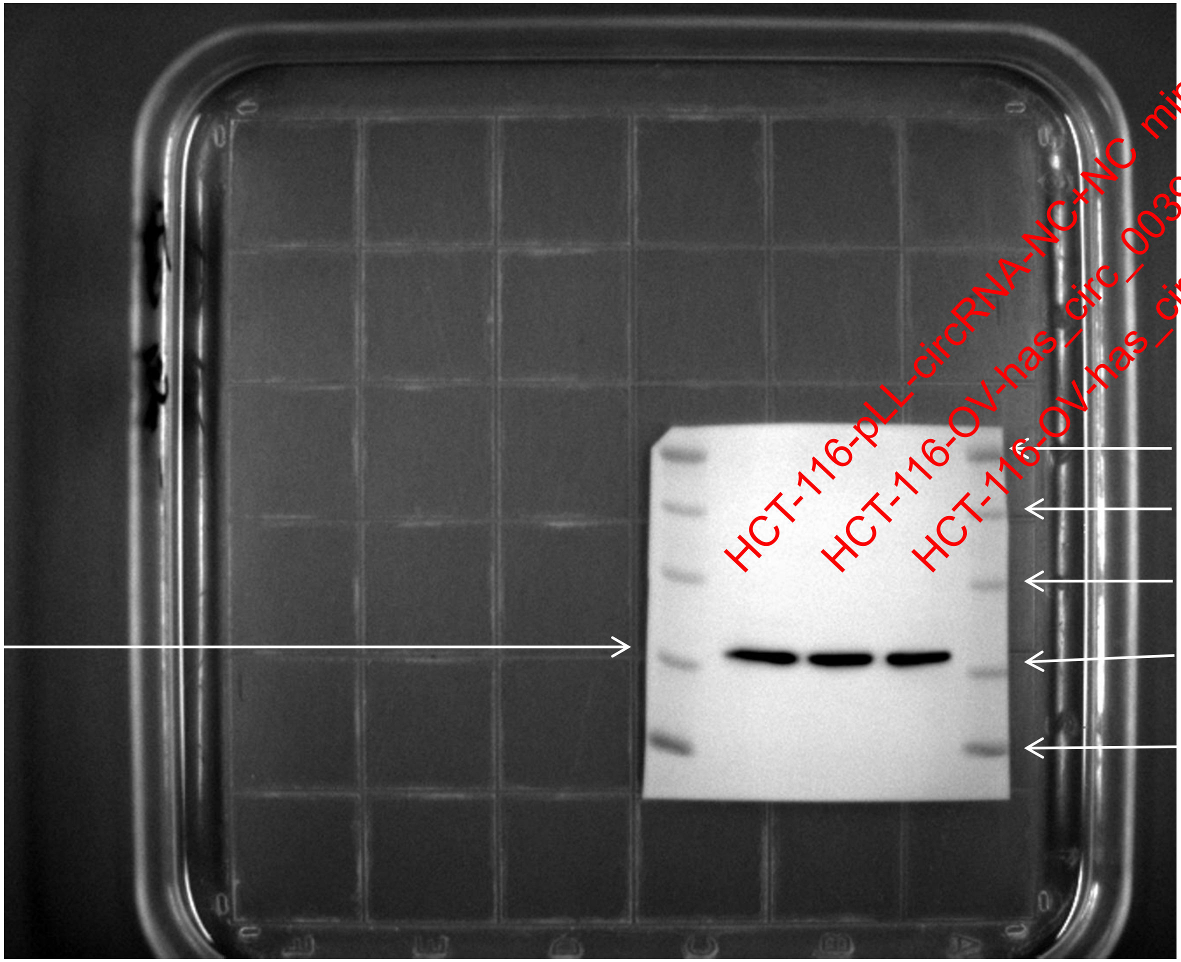

GAPDH, Proteintech, 60004-1-Ig, 1:10000, 36kD;
anti-Mouse IgG, Jackson, 115-035-003, 1:5000

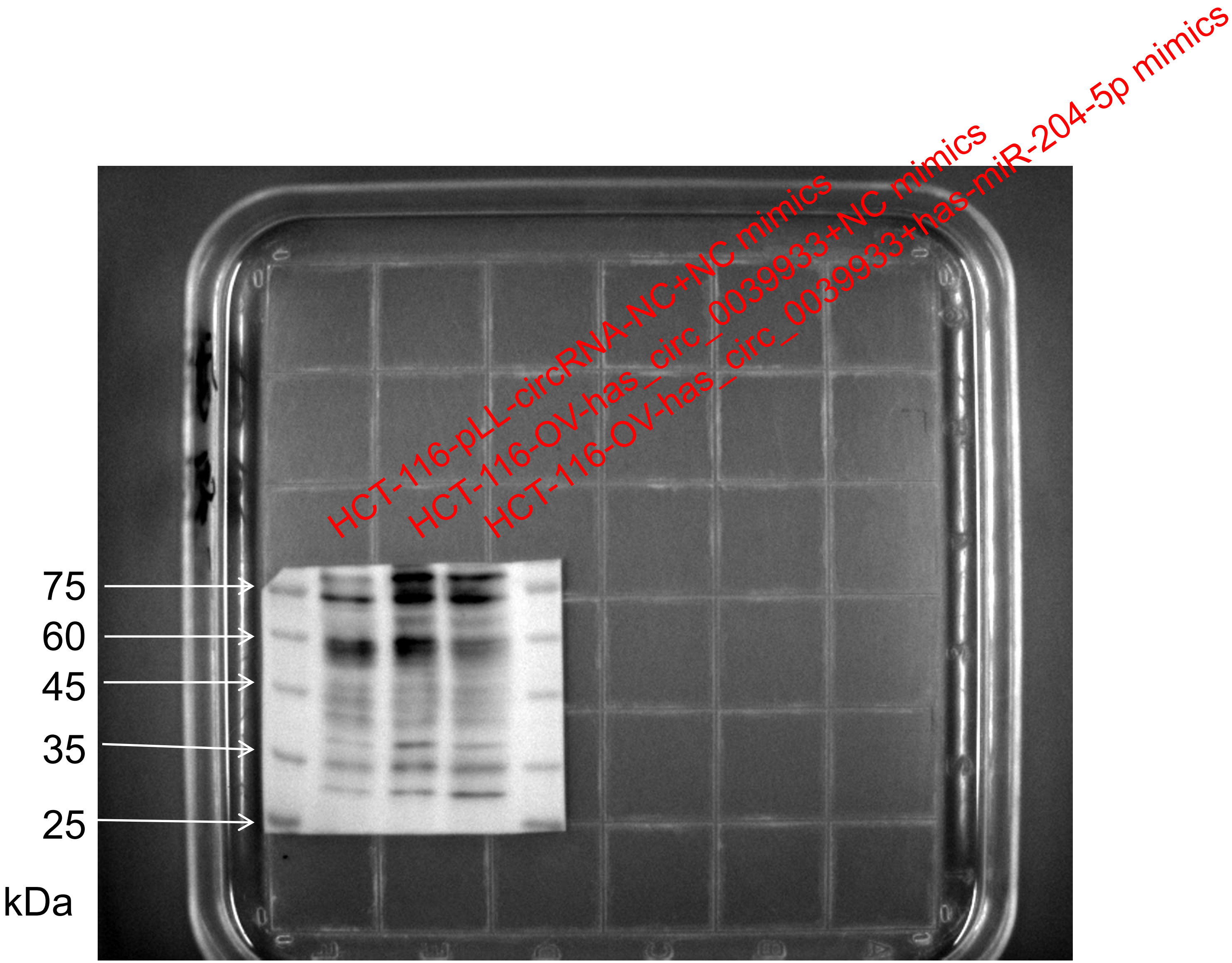

WNT11, Abcam, ab31962, 1:1000, 39kD;
anti-Rabbit IgG, Jackson, 111-035-003, 1:2000

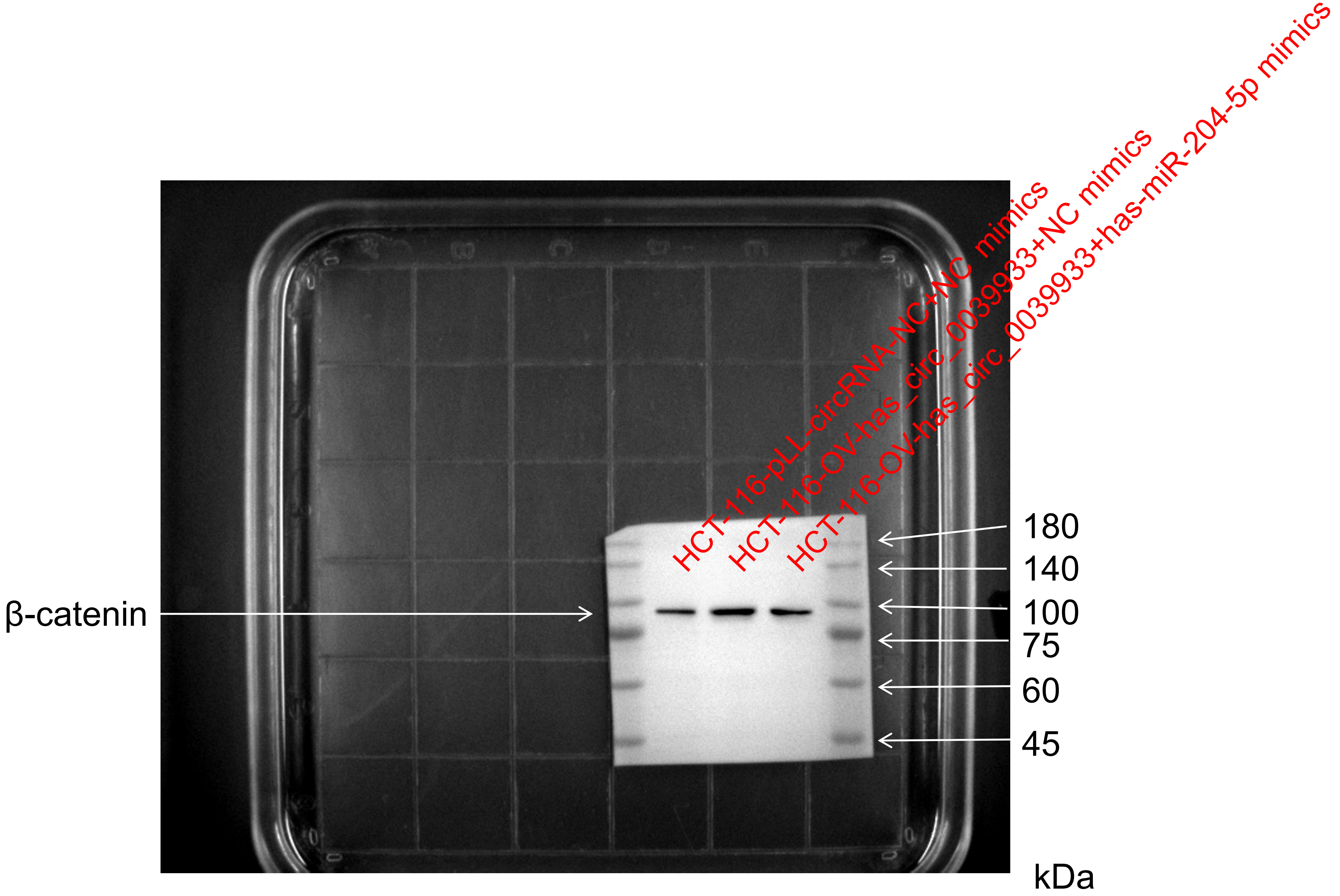

β-catenin, Abcam, ab32572, 1:1000, 92kD;
anti-Rabbit IgG, Jackson, 111-035-003, 1:2000
